# Supplementary material for: Concurrent transmission of Zika virus during the 2023 dengue outbreak in Dhaka, Bangladesh
Source: PLoS Negl Trop Dis. 2025 Jan 30;19(1):e0012866. doi: 10.1371/journal.pntd.0012866 (PMC11813092; doi:10.1371/journal.pntd.0012866)
Supplement: Attachment — Submitted filename: Response to Reviewers.pdf [file pntd.0012866.s001.pdf]

Response to Reviewers

Reviewer 1 Comments:

1. This is explorative study to find ZIKV infection on the dengue study

The objectives of the study are clearly articulated with a clear testable hypothesis stated

The population is clearly described and appropriate for the hypothesis being tested

the study design is appropriate to address the stated objectives

the sample size insufficient to ensure adequate power to address the hypothesis being tested

statistical analysis : NA

Yes they concern about ethical; or regulatory requirement

Response: Thank you for your valuable feedback.

Results

2. This is explorative study to find ZIKV infection on the dengue study, no analysis about Zika

The results is clearly presented

Figures are sufficients

Response: Thank you for your feedback.

3. The strength of the study if teh finding of ZIKV that may impact permanent deficit to the baby

Response: Thank you for your feedback.

Reviewer 2 Comments:

1. Hasan et al., in their manuscript entitled "Concurrent Transmission of Zika Virus During the 2023 Dengue Outbreak in Bangladesh" described 5 cases of Zika during a Dengue epidemic in Bangladesh, one of which was a co-infection of Zika virus and Dengue. The detection of five autochthonous cases of Zika in Bangladesh is a matter of concern considering that the number of countries reporting Zika is increasing worldwide.
In the introduction
line 63, according to ICTV the genus is Orthoflavivirus or use the family Flaviviridae.

Response: It has been corrected.

Results
2. line 179 - Rewrite the sentence better, as it implies that the patients had the same symptom, headache. Of the various symptoms associated with the disease, headache was the only one presented by the 5 cases?

Response: The sentence has been re-written. Yes, headache was the only one symptom presented by all 5 cases.

3. line 185 in the paragraph - Findings from real-time RT-PCR analysis
Needs to make it clearer that only 3 samples were sequenced and justify that "maybe" was because of the high Ct values (low viral load)

Response: The sentence has been re-written accordingly.
4. line 196 - Please rewrite the sentence as it is giving another interpretation. It seems to the reader that this study identified 2 distinct lineages, when in fact the selected ZIKV dataset used sequences representing the two main lineages, Asian and African.

Response: The sentence has been re-written accordingly.
5. line 197- The figure needs to be better presented with this larger phylogenetic tree, plus a detailed description and location of the sequences for better visualization of the data.

Although the phylogenetic tree is well supported and represented, a better presentation of the figure with more enlarged detail should be done to better observe the comments described in the manuscript.

Response: The figure detail has been added more.

Discussion

6. line 217 - Please rewrite the sentence, as it is giving another interpretation. As described in the results, it seems to the reader that this study identified 2 distinct lineages, when in fact the selected ZIKV dataset used sequences representing the two main lineages, Asian and African.

Response: The sentence has been re-written accordingly.

7. line 223 - It should be made clear in this paragraph that although the cases identified were only male and had an average age of 24 years, there is concern about the possibility of possible cases involving children born with congenital anomalies or who suffer premature death, which could have profound socioeconomic and psychosocial consequences in Bangladesh (4, 18)

Response: Thank you for your advice. We have removed the sentence from the manuscript as it has been mentioned in later part as well.

8. line 236 - This information should appear before the paragraph on the line 223 – "As all suspected cases were male, the probability of male-to-female ZIKV transmission and subsequent pre- and peri-natal transmission from mother to descendants would be higher, potentially resulting in the birth of microcephalic neonates (45)."

Response: The sentence has been re-written accordingly.

Reviewer 3 Comments:

1. Introduction

Line 86: The phrase "As far as we know" is too informal; please consider rephrasing it.

Response: It has been rephrased.

2. The title currently says "in Bangladesh," but your sample is only from Dhaka. Does this accurately represent Bangladesh? Consider revising it to "in Dhaka, Bangladesh."

Response: The title has been revised.

3. Were clinical data collected for all participants? You mention in the Introduction (lines 96-97) "corresponding clinical and epidemiological risk factors." If so, it would be beneficial to present and analyze these data with the co-infection or ZIKV infection cases.

Response: Corresponding clinical data has been included in Case report form (CRF) for each case. But we consider only DENV NS1 positive cases for further two follow ups that ultimately curtails necessary clinical data. This sentence has been written as a whole perspective.

4. Line 141: You state that "detecting internal control is not essential." Does this mean you did not use any internal control in your method? If so, please explain why

Response: We mentioned it based on the IFU of the kit we used in this study.

5. From a total of 185 samples meeting the inclusion criteria, you performed RT-PCR on 152 samples. What happened to the remaining 67 samples? Please clarify this in the Methods or Results section. Including a flowchart would make this clearer.

Response: A figure has been given with fishbone diagram.

6. Did you perform any immunological assays? It would be valuable to include immune response data to differentiate past versus current infections and illustrate the situation at the study site.

Response: No, we have not performed any immunological assay so far for those 5 samples. Yes, you are right. It would be valuable if we could include immune response data.

7. The finding of PCR, please add the specific RT-PCR you have done in the sentences, viral load of what virus?

Response: Whole paragraph of finding of PCR has been re-written according to reviewer's advice.

8. If the conclusion in the abstract yes, but please consider to add the conclusion part after the discussion or add in the final paragraph of the discussion, that will be facilitate to add more information rather than only highlight.

Response: A conclusion part has been added.

9. This study was conducted in Dhaka, not throughout Bangladesh, so the sample may not be representative of the entire country. Please consider adding an analysis of clinical data along with the experiments performed, as well as including immunological assays, to make this paper more comprehensive.

Response: Yes, you are right. It is not nationally representative and we mentioned it in our limitation section. We did not do any immunological assays for the time being. But, conducting immunological assays are in our forthcoming research list.
